# Improvements of Jensen-Type Inequalities for Diamond-*α* Integrals

**DOI:** 10.1155/2014/580605

**Published:** 2014-08-14

**Authors:** Rabia Bibi, Josip Pečarić, Mirna Rodić Lipanović

**Affiliations:** ^1^School of Natural Sciences, National University of Sciences and Technology, Islamabad 44000, Pakistan; ^2^Faculty of Textile Technology, University of Zagreb, Prilaz baruna Filipovića 28a, 10000 Zagreb, Croatia

## Abstract

We give further improvements of the Jensen inequality and its converse on time scales, allowing also negative weights. These results generalize the Jensen inequality and its converse for both discrete and continuous cases. Further, we investigate the exponential and logarithmic convexity of the differences between the left-hand side and the right-hand side of these inequalities and present several families of functions for which these results can be applied.

## 1. Preliminaries

The combined dynamic derivative, also called diamond-*α*  (⋄_*α*_) dynamic derivative (*α* ∈ [0,1]), was introduced as a linear convex combination of the well-known delta and nabla dynamic derivatives on time scales. By a time scale *𝕋* we mean any nonempty closed subset of real numbers. Using the delta and nabla derivatives, the notions of delta and nabla integrals were defined (see [1]). We assume, throughout this paper, that the basic notions of the time scales are well known and understood.

**Definition 1 (diamond-*α* integral [1, Definition 3.2]).**

Let *f* : *𝕋* → *ℝ* be continuous function and let *a*, *b* ∈ *𝕋*. Then, the diamond-*α* integral of *f* from *a* to *b* is defined by

(1) ∫abf(t)⋄αt=α∫abf(t)Δt+(1−α)∫abf(t)∇t,aaaaaaaaaaaaaaaaaiaaaaaii0≤α≤1.

**Remark 2 .**

From the above definition it is clear that for *α* = 1 the diamond-*α* integral reduces to the standard delta integral and for *α* = 0 the diamond-*α* integral reduces to the standard nabla integral.

Moreover, if *𝕋* = *ℝ*, then

(2) $$\begin{array}{c} \overset{b}{\underset{a}{\int }}f\left(t\right){⋄}_{\alpha }t=\overset{b}{\underset{a}{\int }}f\left(t\right)\text{d}t; \end{array}$$

if *𝕋* = *ℤ*, then

(3) $$\begin{array}{c} \overset{b}{\underset{a}{\int }}f\left(t\right){⋄}_{\alpha }t=\alpha \overset{b-1}{\underset{t=a}{\sum }}f\left(t\right)+\left(1-\alpha \right)\overset{b}{\underset{t=a+1}{\sum }}f\left(t\right); \end{array}$$

if *𝕋* = *hℤ*, where *h* > 0, then

(4) $$\begin{array}{c} \overset{b}{\underset{a}{\int }}f\left(t\right){⋄}_{\alpha }t=h\left(\alpha \overset{b/h-1}{\underset{k=a/h}{\sum }}f\left(kh\right)+\left(1-\alpha \right)\overset{b/h}{\underset{k=a/h+1}{\sum }}f\left(kh\right)\right); \end{array}$$

if *𝕋* = *q* ^*ℕ*_0_^, where *q* > 1, then

(5) ∫abf(t)⋄αt=(q−1)(α∑k=log⁡q(a)log⁡q(b)−1qkf(qk)aaaaaaiaaa+(1−α)∑k=log⁡q(a)+1log⁡q(b)qk−1f(qk)).

Recently, the Jensen inequality, the improvement of the Jensen inequality, and their converses are given for time scale integrals (see [2–4]).

Let *𝕋* be a time scale, and let *a*, *b* ∈ *𝕋*. Then the time scale interval is denoted and defined by [*a*, *b*]_*𝕋*_ = [*a*, *b*]∩*𝕋*.

**Theorem 3 (see [2]).**

Let *c*, *d* ∈ *ℝ*. If *g* ∈ *C*([*a*, *b*]_*𝕋*_, (*c*, *d*)), *w* ∈ C([*a*, *b*]_*𝕋*_, *ℝ*) with ∫_*a*_^*b*^ | *w*(*t*) | ⋄_*α*_ *t* > 0 and Φ ∈ C((*c*, *d*), *ℝ*) is convex, then

(6) $$\begin{array}{c} \Phi \left(\frac{\int_{a}^{b}g\left(t\right)\left|w\left(t\right)\right|{⋄}_{\alpha }t}{\int_{a}^{b}\left|w\left(t\right)\right|{⋄}_{\alpha }t}\right)\leq \frac{\int_{a}^{b}\Phi \left(g\left(t\right)\right)\left|w\left(t\right)\right|{⋄}_{\alpha }t}{\int_{a}^{b}\left|w\left(t\right)\right|{⋄}_{\alpha }t}. \end{array}$$

Note that in this Jensen inequality we have nonnegative weights. In order to give a better version of this Jensen inequality on time scales, Dinu [4] gives the definition of *α*-Steffensen-Popoviciu (*α*-SP) weight.

**Definition 4 (*α*-SP weight [4, Definition 1]).**

Let *g* ∈ *C*(*𝕋*, *ℝ*). Then, *w* ∈ *C*(*𝕋*, *ℝ*) is an *α*-Steffensen-Popoviciu (*α*-SP) weight for *g* on [*a*, *b*]_*𝕋*_ if

(7) $$\begin{array}{c} \overset{b}{\underset{a}{\int }}w\left(t\right){⋄}_{\alpha }t>0,\;\;\overset{b}{\underset{a}{\int }}\Phi {\left(g\left(t\right)\right)}^{+}w\left(t\right){⋄}_{\alpha }t\geq 0, \end{array}$$

for every convex function Φ ∈ *C*([*m*, *M*], *ℝ*), where

(8) $$\begin{array}{c} m=\underset{t\in [a,b{]}_{T}}{\inf}g\left(t\right),\;\;M=\underset{t\in [a,b{]}_{T}}{\sup}g\left(t\right). \end{array}$$

In the following lemma he gives a characterization for *α*-SP weight for a nondecreasing function *g* on time scales.

**Lemma 5 (see [4, Lemma 2]).**

Let *w* ∈ *C*(*𝕋*, *ℝ*) such that ∫_*a*_^*b*^ *w*(*t*)⋄_*α*_ *t* > 0. Then, *w* is an *α*-SP weight for a nondecreasing function *g* ∈ *C*([*a*, *b*]_*𝕋*_, *ℝ*) if and only if it verifies the following condition:

(9) $$\begin{array}{c} \overset{s}{\underset{a}{\int }}\left(g\left(s\right)-g\left(t\right)\right)w\left(t\right){⋄}_{\alpha }t\geq 0,\\ \overset{b}{\underset{s}{\int }}\left(g\left(t\right)-g\left(s\right)\right)w\left(t\right){⋄}_{\alpha }t\geq 0, \end{array}$$

for every *s* ∈ [*a*, *b*]_*𝕋*_.

If the following stronger (but more suitable) condition holds

(10) $$\begin{array}{c} 0\leq \overset{s}{\underset{a}{\int }}w\left(t\right){⋄}_{\alpha }t\leq \overset{b}{\underset{a}{\int }}w\left(t\right){⋄}_{\alpha }t\;for\;\;every\;\;s\in {\left[a,b\right]}_{T}, \end{array}$$

then *w* is also an *α*-SP weight for the nondecreasing continuous function *g*.

As given in [4], all positive weights are *α*-SP weights, for any continuous function *g* and every *α* ∈ [0,1]. But there are some *α*-SP weights that are allowed to take the negative values. The Jensen inequality on time scales, where it is allowed that the weight function takes some negative values, is given in the following theorem.

**Theorem 6 (see [4, Theorem 2]).**

Let *g* ∈ *C*([*a*, *b*]_*𝕋*_, [*m*, *M*]) and let *w* ∈ *C*([*a*, *b*]_*𝕋*_, *ℝ*) such that ∫_*a*_^*b*^ *w*(*t*)⋄_*α*_ *t* > 0. Then, the following two statements are equivalent:

(i)  
*w* is an *α*-SP weight for *g* on [*a*, *b*]_*𝕋*_;
(ii) * *for every convex function Φ ∈ *C*([*m*, *M*], *ℝ*), it holds
  (11) $$\begin{array}{c} \Phi \left(\frac{\int_{a}^{b}g\left(t\right)w\left(t\right){⋄}_{\alpha }t}{\int_{a}^{b}w\left(t\right){⋄}_{\alpha }t}\right)\leq \frac{\int_{a}^{b}\Phi \left(g\left(t\right)\right)w\left(t\right){⋄}_{\alpha }t}{\int_{a}^{b}w\left(t\right){⋄}_{\alpha }t}. \end{array}$$

**Remark 7 .**

Let *g* be nondecreasing function. If *𝕋* = *ℕ*, then Theorem 6 is equivalent to the Jensen-Steffensen inequality given by Steffensen in [5] (see also [6, page 57]). On the other hand if we take *𝕋* = *ℝ* in Theorem 6, we obtain the integral version of the Jensen-Steffensen inequality given by Boas [7] (see also [6, page 59]).

Considering the converse of the Jensen inequality, Dinu [4] gives the following definition of *α*-Hermite-Hadamard (*α*-HH) weight, its characterization for a nondecreasing function *g* on time scales, and the improvement of the converse of the Jensen inequality for some negative weights.

**Definition 8 (*α*-HH weight [4, Definition 2]).**

Let *g* ∈ *C*(*𝕋*, *ℝ*). Then *w* ∈ *C*(*𝕋*, *ℝ*) is an *α*-Hermite-Hadamard (*α*-HH) weight for *g* on [*a*, *b*]_*𝕋*_ if

(12) $$\begin{array}{c} \overset{b}{\underset{a}{\int }}w\left(t\right){⋄}_{\alpha }t>0,\\ \frac{\int_{a}^{b}\Phi \left(g\left(t\right)\right)w\left(t\right){⋄}_{\alpha }t}{\int_{a}^{b}w\left(t\right){⋄}_{\alpha }t}\leq \frac{M-{\bar{g}}_{\alpha }}{M-m}\Phi \left(m\right)+\frac{{\bar{g}}_{\alpha }-m}{M-m}\Phi \left(M\right), \end{array}$$

for every convex function Φ ∈ *C*([*m*, *M*], *ℝ*), where

(13) m=inf⁡t∈[a,b]Tg(t),  M=sup⁡t∈[a,b]Tg(t),g¯α=∫abg(t)w(t)⋄αt∫abw(t)⋄αt.

**Lemma 9 (see [4, Lemma 3]).**

Let *w* ∈ *C*([*a*, *b*]_*𝕋*_, *ℝ*) be such that ∫_*a*_^*b*^ *w*(*t*)⋄_*α*_ *t* > 0. Then *w* is an *α*-HH weight for a nondecreasing function *g* ∈ *C*([*a*, *b*]_*𝕋*_, *ℝ*) if and only if it verifies the following condition:

(14) g(b)−g(s)g(b)−g(a)∫as(g(t)−g(a))w(t)⋄αt  +g(s)−g(a)g(b)−g(a)∫sb(g(b)−g(t))w(t)⋄αt≥0

for every *s* ∈ [*a*, *b*]_*𝕋*_.

In the next result Dinu [4] gives the connection between these two classes of weights on a time scale.

**Theorem 10 (see [4, Theorem 3]).**

Let *g* ∈ *C*(*𝕋*, *ℝ*). Then every *α*-SP weight for *g* on [*a*, *b*]_*𝕋*_ is an *α*-HH weight for *g* on [*a*, *b*]_*𝕋*_, for all *α* ∈ [0,1].

In the following two sections of our paper we give some further generalizations of the Jensen-type inequalities on time scales allowing negative weights, and we also give the mean-value theorems of the Lagrange and Cauchy type for the functionals obtained by taking the difference of the left-hand side and right-hand side of these new inequalities. These results also generalize the results given in [8] for continuous and discrete cases.  Section 4 in our paper deals with exponential convexity and logarithmic convexity of the functionals obtained in two previous sections. Finally, in Section 5 we present several families of exponentially convex functions which fulfil the conditions of our results. The results from Sections 4 and 5 generalize the results given in [9] for continuous and discrete cases.

## 2. Improvement of the Jensen Inequality on Time Scales

Let *m*, *M* ∈ *ℝ*, where *m* ≠ *M*. Consider the Green function *G* : [*m*, *M*]×[*m*, *M*] → *ℝ* defined by

(15) $$\begin{array}{c} G\left(x,y\right)=\{\begin{array}{cc} \frac{\left(x-M\right)\left(y-m\right)}{M-m} & \text{for}\;\;m\leq y\leq x,\\ \frac{\left(y-M\right)\left(x-m\right)}{M-m} & \text{for}\;\;x\leq y\leq M. \end{array} \end{array}$$

The function *G* is convex and continuous with respect to both *x* and *y*.

It is well known that (see, e.g., [8–11]) any function Φ ∈ *C* ^2^([*m*, *M*], *ℝ*) can be represented by

(16) Φ(x)=M−xM−mΦ(m)+x−mM−mΦ(M)+∫mMG(x,y)Φ′′(y)dy,

where the function *G* is defined in (15). Using (16), we now derive several interesting results concerning the Jensen-type inequalities.

In the following theorem, we give the generalization of the Jensen inequality on time scales, where negative weights are also allowed.

**Theorem 11 .**

Let *g* ∈ *C*([*a*, *b*]_*𝕋*_, *ℝ*) be such that *g*([*a*, *b*]_*𝕋*_)⊆[*m*, *M*]. Let *w* ∈ *C*([*a*, *b*]_*𝕋*_, *ℝ*) be such that ∫_*a*_^*b*^ *w*(*t*)⋄_*α*_ *t* ≠ 0 and ∫_*a*_^*b*^ *g*(*t*)*w*(*t*)⋄_*α*_ *t*/∫_*a*_^*b*^ *w*(*t*)⋄_*α*_ *t* ∈ [*m*, *M*]. Then, the following two statements are equivalent:

(i) for every convex function Φ ∈ *C*([*m*, *M*], *ℝ*)
  (17) $$\begin{array}{c} \Phi \left(\frac{\int_{a}^{b}g\left(t\right)w\left(t\right){⋄}_{\alpha }t}{\int_{a}^{b}w\left(t\right){⋄}_{\alpha }t}\right)\leq \frac{\int_{a}^{b}\Phi \left(g\left(t\right)\right)w\left(t\right){⋄}_{\alpha }t}{\int_{a}^{b}w\left(t\right){⋄}_{\alpha }t} \end{array}$$
  holds;
(ii) for all *y* ∈ [*m*, *M*]
  (18) $$\begin{array}{c} G\left(\frac{\int_{a}^{b}g\left(t\right)w\left(t\right){⋄}_{\alpha }t}{\int_{a}^{b}w\left(t\right){⋄}_{\alpha }t},y\right)\leq \frac{\int_{a}^{b}G\left(g\left(t\right),y\right)w\left(t\right){⋄}_{\alpha }t}{\int_{a}^{b}w\left(t\right){⋄}_{\alpha }t} \end{array}$$
  holds, where *G* : [*m*, *M*]×[*m*, *M*] → *ℝ* is defined in (15).

Furthermore, the statements (i) and (ii) are also equivalent if we change the sign of inequality in both (17) and (18).

**Proof**

(i) ⇒ (ii): let (i) hold. As the function *G*(·, *y*) (*y* ∈ [*m*, *M*]) is continuous and convex, it follows that (18) holds.

(ii) ⇒ (i): let (ii) hold. Let Φ ∈ *C* ^2^([*m*, *M*], *ℝ*). Then by using (16) we get

(19) ∫abΦ(g(t))w(t)⋄αt∫abw(t)⋄αt−Φ(∫abg(t)w(t)⋄αt∫abw(t)⋄αt) =∫mM[∫abG(g(t),y)w(t)⋄αt∫abw(t)⋄αtaaaaaaaii− G(∫abg(t)w(t)⋄αt∫abw(t)⋄αt,y)]Φ′′(y)dy.

If the function Φ is also convex, then Φ′′(*y*) ≥ 0 for all *y* ∈ [*m*, *M*], and hence it follows that for every convex function Φ ∈ *C* ^2^([*m*, *M*], *ℝ*) inequality (17) holds. Moreover, it is not necessary to demand the existence of the second derivative of the function Φ (see [6, page 172]). The differentiability condition can be directly eliminated by using the fact that it is possible to approximate uniformly a continuous convex function by convex polynomials.

The last part of our theorem can be proved analogously.

**Remark 12 .**

Let the conditions of Theorem 11 hold. Then the following two statements are equivalent.

(${\text{i}}^{\prime }$) For every concave function Φ ∈ *C*([*m*, *M*], *ℝ*) the reverse inequality in (17) holds.
(${\text{ii}}^{\prime }$) For all *y* ∈ [*m*, *M*] inequality (18) holds.

Moreover, statements (i′) and (ii′) are also equivalent if we change the sign of inequality in both statements (i′) and (ii′).

**Remark 13 .**

Consider (19). Suppose that *g* is nondecreasing and that it has the first derivative. Let *m* = *g*(*a*) and *M* = *g*(*b*), and make the substitution *y* = *g*(*s*). Then we get

(20) ∫abΦ(g(t))w(t)⋄αt∫abw(t)⋄αt−Φ(∫abg(t)w(t)⋄αt∫abw(t)⋄αt) =∫ab[∫abG(g(t),g(s))w(t)⋄αt∫abw(t)⋄αtaaaaaaai− G(∫abg(t)w(t)⋄αt∫abw(t)⋄αt,g(s))]aaaaaaai×Φ′′(g(s))g′(s)ds.

Since *g* is nondecreasing, we have that *g*′(*s*) ≥ 0 for all *s* ∈ [*a*, *b*]_*𝕋*_. If Φ ∈ *C* ^2^([*m*, *M*], *ℝ*) is convex, then Φ′′(*g*(s)) ≥ 0 for all *s* ∈ [*a*, *b*]_*𝕋*_. Hence, if and only if

(21) $$\begin{array}{c} G\left(\frac{\int_{a}^{b}g\left(t\right)w\left(t\right){⋄}_{\alpha }t}{\int_{a}^{b}w\left(t\right){⋄}_{\alpha }t},g\left(s\right)\right)\leq \frac{\int_{a}^{b}G\left(g\left(t\right),g\left(s\right)\right)w\left(t\right){⋄}_{\alpha }t}{\int_{a}^{b}w\left(t\right){⋄}_{\alpha }t} \end{array}$$

holds for all *s* ∈ [*a*, *b*]_*𝕋*_, then for every continuous convex function Φ inequality (17) holds.

Combining the result from Theorem 11 with Theorem 6 and Lemma 5, we get the following two corollaries.

**Corollary 14 .**

Let *g* ∈ *C*([*a*, *b*]_*𝕋*_, [*m*, *M*]) and let *w* ∈ *C*([*a*, *b*]_*𝕋*_, *ℝ*) be such that ∫_*a*_^*b*^ *w*(*t*)⋄_*α*_ *t* > 0. Then *w* is an *α*-SP weight for *g* on [*a*, *b*]_*𝕋*_ if and only if

(22) $$\begin{array}{c} G\left(\frac{\int_{a}^{b}g\left(t\right)w\left(t\right){⋄}_{\alpha }t}{\int_{a}^{b}w\left(t\right){⋄}_{\alpha }t},y\right)\leq \frac{\int_{a}^{b}G\left(g\left(t\right),y\right)w\left(t\right){⋄}_{\alpha }t}{\int_{a}^{b}w\left(t\right){⋄}_{\alpha }t} \end{array}$$

holds for all *y* ∈ [*m*, *M*], where *G* is defined in (15).

**Corollary 15 .**

Let *g* ∈ *C*([*a*, *b*]_*𝕋*_, [*m*, *M*]) be nondecreasing function and *w* ∈ *C*([*a*, *b*]_*𝕋*_, *ℝ*) such that ∫_*a*_^*b*^ *w*(*t*)⋄_*α*_ *t* > 0. Then

(23) $$\begin{array}{c} \overset{s}{\underset{a}{\int }}\left(g\left(s\right)-g\left(t\right)\right)w\left(t\right){⋄}_{\alpha }t\geq 0,\\ \overset{b}{\underset{s}{\int }}\left(g\left(t\right)-g\left(s\right)\right)w\left(t\right){⋄}_{\alpha }t\geq 0 \end{array}$$

hold for all *s* ∈ [*a*, *b*]_*𝕋*_, if and only if

(24) $$\begin{array}{c} G\left(\frac{\int_{a}^{b}g\left(t\right)w\left(t\right){⋄}_{\alpha }t}{\int_{a}^{b}w\left(t\right){⋄}_{\alpha }t},y\right)\leq \frac{\int_{a}^{b}G\left(g\left(t\right),y\right)w\left(t\right){⋄}_{\alpha }t}{\int_{a}^{b}w\left(t\right){⋄}_{\alpha }t} \end{array}$$

holds for all *y* ∈ [*m*, *M*], where *G* is defined in (15).

To shorten the notation, in the sequel we will use the following notation:

(25) $$\begin{array}{c} {\bar{g}}_{\alpha }=\frac{\int_{a}^{b}g\left(t\right)w\left(t\right){⋄}_{\alpha }t}{\int_{a}^{b}w\left(t\right){⋄}_{\alpha }t}. \end{array}$$

Under the assumptions of Theorem 11, we define the following functional *𝒥*_1_(*g*, Φ):

(26) J1(g,Φ)={∫abΦ(g(t))w(t)⋄αt∫abw(t)⋄αt−Φ(∫abg(t)w(t)⋄αt∫abw(t)⋄αt)  if  ∀y∈[m,M]  inequality  (18)  holds,Φ(∫abg(t)w(t)⋄αt∫abw(t)⋄αt)−∫abΦ(g(t))w(t)⋄αt∫abw(t)⋄αt  if  ∀y∈[m,M]  the  reverse  inequality in  (18)  holds,

where the function Φ is defined on [*m*, *M*]. Clearly, if Φ is continuous and convex, then *𝒥*_1_(*g*, Φ) is nonnegative.

**Theorem 16 .**

Let *g* ∈ *C*([*a*, *b*]_*𝕋*_, *ℝ*) and Φ ∈ *C* ^2^([*m*, *M*], *ℝ*). Let *𝒥*_1_ be defined as in (26). Then there exists *ξ* ∈ [*m*, *M*] such that

(27) $$\begin{array}{c} J_{1}\left(g,\Phi \right)=\Phi^{\prime \prime }\left(\xi \right)J_{1}\left(g,\Phi_{0}\right) \end{array}$$

holds, where Φ_0_(*x*) = *x* ^2^/2.

**Proof**

Since the function Φ′′ is continuous and

(28) $$\begin{array}{c} \frac{\int_{a}^{b}G\left(g\left(t\right),y\right)w\left(t\right){⋄}_{\alpha }t}{\int_{a}^{b}w\left(t\right){⋄}_{\alpha }t}-G\left(\frac{\int_{a}^{b}g\left(t\right)w\left(t\right){⋄}_{\alpha }t}{\int_{a}^{b}w\left(t\right){⋄}_{\alpha }t},y\right) \end{array}$$

does not change its positivity on [*m*, *M*], applying the integral mean-value theorem on (19) we get that there exists *ξ* ∈ [*m*, *M*] such that

(29) ∫abΦ(g(t))w(t)⋄αt∫abw(t)⋄αt−Φ(∫abg(t)w(t)⋄αt∫abw(t)⋄αt) =Φ′′(ξ)∫mM[∫abG(g(t),y)w(t)⋄αt∫abw(t)⋄αtaaaaaaaaaaaaaai− G(∫abg(t)w(t)⋄αt∫abw(t)⋄αt,y)]dy

holds. As in [12], it can be easily checked that it holds

(30) ∫mMG(x,y)dy=∫mx(x−M)(y−m)M−mdy +∫xM(y−M)(x−m)M−mdy=12(x−m)(x−M).

Calculating the integral on the right-hand side of (29), we get

(31) ∫abΦ(g(t))w(t)⋄αt∫abw(t)⋄αt−Φ(∫abg(t)w(t)⋄αt∫abw(t)⋄αt) =Φ′′(ξ)[1∫abw(t)⋄αtaaaaaaaaiiaa×∫ab(∫mMG(g(t),y)dy)w(t)⋄αtaaaaaaaaaaa−∫mMG(g¯α,y)dy1∫abw(t)⋄αt] =Φ′′(ξ)[1∫abw(t)⋄αt∫ab12(g(t)−m)aaaaaaaaaaaaaaaaaaaaaai×(g(t)−M)w(t)⋄αtaaaaaaaaaai−12(g¯α−m)(g¯α−M)1∫abw(t)⋄αt] =12Φ′′(ξ)[∫ab(g(t))2w(t)⋄αt∫abw(t)⋄αt−g¯α2]

and the proof is completed.

**Remark 17 .**

Theorem 16 can also be proved by using the following two convex functions:

(32) $$\begin{array}{c} \phi_{1}\left(x\right)=\frac{\zeta }{2}x^{2}-\Phi \left(x\right),\;\;\phi_{2}\left(x\right)=\Phi \left(x\right)-\frac{\eta }{2}x^{2}, \end{array}$$

where

(33) $$\begin{array}{c} \eta =\underset{x\in [m,M]}{\min}\Phi^{\prime \prime }\left(x\right),\;\;\zeta =\underset{x\in [m,M]}{\max}\Phi^{\prime \prime }\left(x\right). \end{array}$$

Since *ϕ*_1_ and *ϕ*_2_ are continuous and convex, we have

(34) $$\begin{array}{c} J_{1}\left(g,\phi_{1}\right)\geq 0,\;\;J_{1}\left(g,\phi_{2}\right)\geq 0. \end{array}$$

This implies that

(35) $$\begin{array}{c} \eta J_{1}\left(g,\Phi_{0}\right)\leq J_{1}\left(g,\Phi \right)\leq \zeta J_{1}\left(g,\Phi_{0}\right). \end{array}$$

Hence, as the function Φ′′ is continuous, there exists *ξ* ∈ [*m*, *M*] such that (27) holds.

**Theorem 18 .**

Let *g* ∈ *C*([*a*, *b*]_*𝕋*_, *ℝ*) and Φ, Ψ ∈ *C* ^2^([*m*, *M*], *ℝ*). Let *𝒥*_1_ be defined as in (26). Then there exists *ξ* ∈ [*m*, *M*] such that

(36) $$\begin{array}{c} \frac{J_{1}\left(g,\Phi \right)}{J_{1}\left(g,\Psi \right)}=\frac{\Phi^{\prime \prime }\left(\xi \right)}{\Psi^{\prime \prime }\left(\xi \right)} \end{array}$$

holds, provided that the denominator on the left-hand side of (36) is nonzero.

**Proof**

Let *χ* be defined as the linear combination of functions Φ and Ψ by

(37) $$\begin{array}{c} \chi \left(x\right)=J_{1}\left(g,\Psi \right)\Phi \left(x\right)-J_{1}\left(g,\Phi \right)\Psi \left(x\right). \end{array}$$

Then *χ* ∈ *C* ^2^([*m*, *M*], *ℝ*). By applying Theorem 16 on *χ*, it follows that there exists *ξ* ∈ [*m*, *M*] such that

(38) $$\begin{array}{c} J_{1}\left(g,\chi \right)=\chi^{\prime \prime }\left(\xi \right)J_{1}\left(g,\Phi_{0}\right). \end{array}$$

After a short calculation we get that *𝒥*_1_(*g*, *χ*) = 0. By hypothesis *𝒥*_1_(*g*, Φ_0_) ≠ 0 (otherwise, we have a contradiction with *𝒥*_1_(*g*, Ψ) ≠ 0), so it follows that

(39) $$\begin{array}{c} \chi^{\prime \prime }\left(\xi \right)=0, \end{array}$$

which is equivalent to (36).

**Remark 19 .**

In Theorem 18, if the inverse of the function Φ′′/Ψ′′ exists, then (36) gives

(40) $$\begin{array}{c} \xi ={\left(\frac{\Phi^{\prime \prime }}{\Psi^{\prime \prime }}\right)}^{-1}\left(\frac{J_{1}\left(g,\Phi \right)}{J_{1}\left(g,\Psi \right)}\right). \end{array}$$

**Remark 20 .**

Note that setting the function Ψ as Ψ(*x*) = *x* ^2^/2 in Theorem 18, we get the statement of Theorem 16.

As a consequence of the above two mean-value theorems, the following corollaries easily follow.

**Corollary 21 .**

Let *g* ∈ *C*([*a*, *b*]_*𝕋*_, [*m*, *M*]), Φ, Ψ : [*m*, *M*] → *ℝ*, and let *w* ∈ *C*([*a*, *b*]_*𝕋*_, *ℝ*) be an *α*-SP weight for *g*. Let *𝒥*_1_ be defined as in (26). Then the following two statements hold.

(i) If Φ ∈ *C* ^2^([*m*, *M*], *ℝ*), then there exists *ξ* ∈ [*m*, *M*] such that (27) holds.
(ii) If Φ, Ψ ∈ *C* ^2^([*m*, *M*], *ℝ*), then there exists *ξ* ∈ [*m*, *M*] such that (36) holds.

**Proof**

The statement (i) (statement (ii), resp.,) directly follows from Theorem 16 (Theorem 18, resp.,) and Corollary 14.

**Corollary 22 .**

Let *g* ∈ *C*([*a*, *b*]_*𝕋*_, [*m*, *M*]) be monotone function, Φ, Ψ : [*m*, *M*] → *ℝ*, and *w* ∈ *C*([*a*, *b*]_*𝕋*_, *ℝ*) such that ∫_*a*_^*b*^ *w*(*t*)⋄_*α*_ *t* > 0. Let (9) hold for all *s* ∈ [*a*, *b*]_*𝕋*_. Let *𝒥*_1_ be defined as in (26). Then the following two statements hold.

(i) If Φ ∈ *C* ^2^([*m*, *M*], *ℝ*), then there exists *ξ* ∈ [*m*, *M*] such that (27) holds.
(ii) If Φ, Ψ ∈ *C* ^2^([*m*, *M*], *ℝ*), then there exists *ξ* ∈ [*m*, *M*] such that (36) holds.

**Proof**

The statement (i) (statement (ii), resp.,) directly follows from Theorem 16 (Theorem 18, resp.,) and Corollary 15.

## 3. Improvement of the Converse of the Jensen Inequality on Time Scales

Using the similar method as in previous section, in the following theorem we obtain the generalization of the converse of the Jensen inequality on time scales, where negative weights are also allowed.

**Theorem 23 .**

Let *g* ∈ *C*([*a*, *b*]_*𝕋*_, *ℝ*) be such that *g*([*a*, *b*]_*𝕋*_)⊆[*m*, *M*] and let *c*, *d* ∈ [*m*, *M*]  (*c* ≠ *d*) be such that *c* ≤ *g*(*t*) ≤ *d* for all *t* ∈ [*a*, *b*]_*𝕋*_. Let *w* ∈ *C*([*a*, *b*]_*𝕋*_, *ℝ*) be such that ∫_*a*_^*b*^ *w*(*t*)⋄_*α*_ *t* ≠ 0. Then, the following two statements are equivalent.

(i) For every convex function Φ ∈ *C*([*m*, *M*], *ℝ*)
  (41) $$\begin{array}{c} \frac{\int_{a}^{b}\Phi \left(g\left(t\right)\right)w\left(t\right){⋄}_{\alpha }t}{\int_{a}^{b}w\left(t\right){⋄}_{\alpha }t}\leq \frac{d-{\bar{g}}_{\alpha }}{d-c}\Phi \left(c\right)+\frac{{\bar{g}}_{\alpha }-c}{d-c}\Phi \left(d\right) \end{array}$$
  holds.
(ii) For all *y* ∈ [*m*, *M*]
  (42) $$\begin{array}{c} \frac{\int_{a}^{b}G\left(g\left(t\right),y\right)w\left(t\right){⋄}_{\alpha }t}{\int_{a}^{b}w\left(t\right){⋄}_{\alpha }t}\leq \frac{d-{\bar{g}}_{\alpha }}{d-c}G\left(c,y\right)+\frac{{\bar{g}}_{\alpha }-c}{d-c}G\left(d,y\right) \end{array}$$
  holds, where the function *G* : [*m*, *M*]×[*m*, *M*] → *ℝ* is defined in (15).

Furthermore, the statements (i) and (ii) are also equivalent if we change the sign of inequality in both (41) and (42).

**Proof**

The idea of the proof is very similar to the proof of Theorem 11.

(i) ⇒ (ii): let (i) hold. As the function *G*(·, *y*) (*y* ∈ [*m*, *M*]) is continuous and convex, it follows that (42) holds.

(ii) ⇒ (i): let (ii) hold. Let Φ ∈ *C* ^2^([*m*, *M*], *ℝ*). Then by using (16) we get

(43) d−g¯αd−cΦ(c)+g¯α−cd−cΦ(d)−∫abΦ(g(t))w(t)⋄αt∫abw(t)⋄αt =∫mM[∫abG(g(t),y)w(t)⋄αt∫abw(t)⋄αtd−g¯αd−cG(c,y)+g¯α−cd−cG(d,y)aaaaaaaa− ∫abG(g(t),y)w(t)⋄αt∫abw(t)⋄αt]Φ′′(y)dy.

If the function Φ is also convex, then Φ′′(*y*) ≥ 0 for all *y* ∈ [*m*, *M*], and hence it follows that for every convex function Φ ∈ *C* ^2^([*m*, *M*], *ℝ*) the inequality (41) holds. Moreover, it is not necessary to demand the existence of the second derivative of the function Φ (see [6, page 172]). The differentiability condition can be directly eliminated by using the fact that it is possible to approximate uniformly a continuous convex function by convex polynomials.

The last part of our theorem can be proved analogously.

**Remark 24 .**

Let the conditions of Theorem 23 hold. Then the following two statements are equivalent.

(${\text{i}}^{\prime }$) For every concave function Φ ∈ *C*([*m*, *M*], *ℝ*), the reverse inequality in (41) holds.
(${\text{ii}}^{\prime }$) For all *y* ∈ [*m*, *M*] inequality (42) holds.

Moreover, statements (i′) and (ii′) are also equivalent if we change the sign of inequality in both statements (i′) and (ii′).

**Remark 25 .**

Note that in all the results in this section we allow that the mean value ${\bar{g}}_{\alpha }$ goes out of the interval [*m*, *M*], while in the results from the previous section we demanded that ${\bar{g}}_{\alpha }\in [m,M]$.

Setting *c* = *m* and *d* = *M* in Theorem 23, we get the following result.

**Corollary 26 .**

Let *g* ∈ *C*([*a*, *b*]_*𝕋*_, *ℝ*) be such that *g*([*a*, *b*]_*𝕋*_)⊆[*m*, *M*]. Let *w* ∈ *C*([*a*, *b*]_*𝕋*_, *ℝ*) be such that ∫_*a*_^*b*^ *w*(*t*)⋄_*α*_ *t* ≠ 0. Then, the following two statements are equivalent.

(i) For every convex function Φ ∈ *C*([*m*, *M*], *ℝ*)
  (44) $$\begin{array}{c} \frac{\int_{a}^{b}\Phi \left(g\left(t\right)\right)w\left(t\right){⋄}_{\alpha }t}{\int_{a}^{b}w\left(t\right){⋄}_{\alpha }t}\leq \frac{M-{\bar{g}}_{\alpha }}{M-m}\Phi \left(m\right)+\frac{{\bar{g}}_{\alpha }-m}{M-m}\Phi \left(M\right) \end{array}$$
  holds.
(ii) For all *y* ∈ [*m*, *M*]
  (45) $$\begin{array}{c} \frac{\int_{a}^{b}G\left(g\left(t\right),y\right)w\left(t\right){⋄}_{\alpha }t}{\int_{a}^{b}w\left(t\right){⋄}_{\alpha }t}\leq 0 \end{array}$$
  holds, where the function *G* : [*m*, *M*]×[*m*, *M*] → *ℝ* is defined as in (15).

Furthermore, statements (i) and (ii) are also equivalent if we change the sign of inequality in both (44) and (45).

**Remark 27 .**

As a consequence of Corollary 26, we get the result from Lemma 9.

Let *c* = *m* and *d* = *M*. Then (43) transforms into

(46) M−g¯αM−mΦ(m)+g¯α−mM−mΦ(M)−∫abΦ(g(t))w(t)⋄αt∫abw(t)⋄αt =−∫mM∫abG(g(t),y)w(t)⋄αt∫abw(t)⋄αtΦ′′(y)dy.

Let ∫_*a*_^*b*^ *w*(*t*)⋄_*α*_ *t* > 0, and suppose that *g* is nondecreasing and that it has the first derivative. Now, similarly as in [4], we derive the result from Lemma 9. Let *m* = *g*(*a*) and *M* = *g*(*b*), and make the substitution *y* = *g*(*s*). Then we get

(47) M−g¯αM−mΦ(m)+g¯α−mM−mΦ(M)−∫abΦ(g(t))w(t)⋄αt∫abw(t)⋄αt =−1∫abw(t)⋄αt∫ab(∫abG(g(t),g(s))w(t)⋄αt)aaaaaaaaaaaaaaaaa×Φ′′(g(s))g′(s)ds.

Since *g* is nondecreasing, we have that *g*′(*s*) ≥ 0 for all *s* ∈ [*a*, *b*]_*𝕋*_. If Φ ∈ *C* ^2^([*m*, *M*], *ℝ*) is convex, then Φ′′(*g*(*s*)) ≥ 0 for all *s* ∈ [*a*, *b*]_*𝕋*_. Hence, if and only if

(48) $$\begin{array}{c} \overset{b}{\underset{a}{\int }}G\left(g\left(t\right),g\left(s\right)\right)w\left(t\right){⋄}_{\alpha }t\leq 0 \end{array}$$

holds for all *s* ∈ [*a*, *b*]_*𝕋*_, then for every continuous convex function Φ inequality (44) holds. Note that (48) is equivalent to condition (14).

**Corollary 28 .**

Let *g* ∈ *C*([*a*, *b*]_*𝕋*_, [*m*, *M*]) and let *w* ∈ *C*([*a*, *b*]_*𝕋*_, *ℝ*) be an *α*-SP weight for *g* on [*a*, *b*]_*𝕋*_. Then

(49) $$\begin{array}{c} \frac{\int_{a}^{b}G\left(g\left(t\right),y\right)w\left(t\right){⋄}_{\alpha }t}{\int_{a}^{b}w\left(t\right){⋄}_{\alpha }t}\leq 0 \end{array}$$

holds for all *y* ∈ [*m*, *M*].

**Proof**

The proof follows directly from Theorem 10 and Corollary 26.

Under the assumptions of Theorem 23, we define the following functional *𝒥*_2_(*g*, Φ):

(50) J2(g,Φ)={d−g¯αd−cΦ(c)+g¯α−cd−cΦ(d)−∫abΦ(g(t))w(t)⋄αt∫abw(t)⋄αtaaaaaaaif  ∀y∈[m,M]  inequality  (42)  holds,∫abΦ(g(t))w(t)⋄αt∫abw(t)⋄αt−d−g¯αd−cΦ(c)−g¯α−cd−cΦ(d)aaaaaaaif  ∀y∈[m,M]  the  reverse  inequality  aaaaaaain  (42)  holds,

where the function Φ is defined on [*m*, *M*]. Clearly, if Φ is continuous and convex, then *𝒥*_2_(*g*, Φ) is nonnegative.

**Theorem 29 .**

Let *g* ∈ *C*([*a*, *b*]_*𝕋*_, *ℝ*) and Φ ∈ *C* ^2^([*m*, *M*], *ℝ*). Let *𝒥*_2_ be defined as in (50). Then there exists *ξ* ∈ [*m*, *M*] such that

(51) $$\begin{array}{c} J_{2}\left(g,\Phi \right)=\Phi^{\prime \prime }\left(\xi \right)J_{2}\left(g,\Phi_{0}\right) \end{array}$$

holds, where Φ_0_(*x*) = *x* ^2^/2.

**Proof**

The idea of the proof is very similar to the proof of Theorem 16.

Since the function Φ′′ is continuous and

(52) $$\begin{array}{c} \frac{d-{\bar{g}}_{\alpha }}{d-c}G\left(c,y\right)+\frac{{\bar{g}}_{\alpha }-c}{d-c}G\left(d,y\right)-\frac{\int_{a}^{b}G\left(g\left(t\right),y\right)w\left(t\right){⋄}_{\alpha }t}{\int_{a}^{b}w\left(t\right){⋄}_{\alpha }t} \end{array}$$

does not change its positivity on [*m*, *M*], applying the integral mean-value theorem on (43) we get that there exists *ξ* ∈ [*m*, *M*] such that

(53) d−g¯αd−cΦ(c)+g¯α−cd−cΦ(d)−∫abΦ(g(t))w(t)⋄αt∫abw(t)⋄αt =Φ′′(ξ)∫mM[∫abG(g(t),y)w(t)⋄αt∫abw(t)⋄αtd−g¯αd−cG(c,y)+g¯α−cd−cG(d,y)aaaaaaaaaaaaai− ∫abG(g(t),y)w(t)⋄αt∫abw(t)⋄αt]dy.

Calculating the integral on the right-hand side of (53), we get

(54) d−g¯αd−cΦ(c)+g¯α−cd−cΦ(d)−∫abΦ(g(t))w(t)⋄αt∫abw(t)⋄αt =12Φ′′(ξ)[∫ab(g(t))2w(t)⋄αt∫abw(t)⋄αtd−g¯αd−c·c2+g¯α−cd−c·d2aaaaaaaaaaaa− ∫ab(g(t))2w(t)⋄αt∫abw(t)⋄αt]

and we get statement (51) of our theorem.

**Remark 30 .**

Note that (54) can also be expressed as

(55) d−g¯αd−cΦ(c)+g¯α−cd−cΦ(d)−∫abΦ(g(t))w(t)⋄αt∫abw(t)⋄αt =12Φ′′(ξ)[g¯α(c+d)−cd−∫ab(g(t))2w(t)⋄αt∫abw(t)⋄αt].

**Theorem 31 .**

Let *g* ∈ *C*([*a*, *b*]_*𝕋*_, *ℝ*) and Φ, Ψ ∈ *C* ^2^([*m*, *M*], *ℝ*). Let *𝒥*_2_ be defined as in (50). Then there exists *ξ* ∈ [*m*, *M*] such that

(56) $$\begin{array}{c} \frac{J_{2}\left(g,\Phi \right)}{J_{2}\left(g,\Psi \right)}=\frac{\Phi^{\prime \prime }\left(\xi \right)}{\Psi^{\prime \prime }\left(\xi \right)} \end{array}$$

holds, provided that the denominator on the left-hand side of (56) is nonzero.

**Proof**

The proof is very similar to the proof of Theorem 18.

## 4. Exponential and Logarithmic Convexity

First we recall some definitions and facts about exponentially convex and logarithmically convex functions (see, e.g., [13, 14] or [9]) which we need for our results.

**Definition 32 .**

A function *f* : *I* → *ℝ*(*I*⊆*ℝ*) is *n*-exponentially convex in the Jensen sense on *I*, if

(57) $$\begin{array}{c} \overset{n}{\underset{i,j=1}{\sum }}\xi_{i}\xi_{j}f\left(\frac{x_{i}+x_{j}}{2}\right)\geq 0 \end{array}$$

holds for all choices *ξ*_*i*_ ∈ *ℝ* and *x*_*i*_ ∈ *I*, *i* = 1,…, *n*.

A function *f* : *I* → *ℝ* is *n*-exponentially convex if it is *n*-exponentially convex in the Jensen sense and continuous on *I*.

**Remark 33 .**

It is clear from the definition that 1-exponentially convex functions in the Jensen sense are in fact nonnegative functions. Also, *n*-exponentially convex functions in the Jensen sense are *k*-exponentially convex in the Jensen sense for every *k* ∈ *ℕ*, *k* ≤ *n*.

By definition of positive semidefinite matrices and some basic linear algebra, we have the following proposition.

**Proposition 34 .**

If *f* is an *n*-exponentially convex function in the Jensen sense, then the matrix [*f*((*x*_*i*_ + *x*_*j*_)/2)]_*i*,*j*=1_^*k*^ is positive, semidefinite for all *k* ∈ *ℕ*, *k* ≤ *n*. Particularly, det⁡[*f*((*x*_*i*_ + *x*_*j*_)/2)]_*i*,*j*=1_^*k*^ ≥ 0 for all *k* ∈ *ℕ*, *k* ≤ *n*.

**Definition 35 .**

A function *f* : *I* → *ℝ* is exponentially convex in the Jensen sense on *I*, if it is *n*-exponentially convex in the Jensen sense for all *n* ∈ *ℕ*.

A function *f* : *I* → *ℝ* is exponentially convex if it is exponentially convex in the Jensen sense and continuous.

**Remark 36 .**

Some examples of exponentially convex functions are (see [15]) as follows:

(i) *f* : *I* → *ℝ* defined by *f*(*x*) = *ce* ^*kx*^, where *c* ≥ 0 and *k* ∈ *ℝ*;
(ii) *f* : *ℝ* ^+^ → *ℝ* defined by *f*(*x*) = *x* ^−*k*^, where *k* > 0;
(iii) *f* : *ℝ* ^+^ → *ℝ* ^+^ defined by $f(x)=e^{-k\sqrt{x}}$, where *k* > 0.

**Remark 37 .**

It is known (and easy to show) that *f* : *I* → *ℝ* ^+^ is log-convex in the Jensen sense on *I*, if and only if

(58) $$\begin{array}{c} \rho^{2}f\left(x\right)+2\rho \sigma f\left(\frac{x+y}{2}\right)+\sigma^{2}f\left(y\right)\geq 0 \end{array}$$

holds for every *ρ*, *σ* ∈ *ℝ* and *x*, *y* ∈ *I*. It follows that a positive function is log-convex in the Jensen sense if and only if it is 2-exponentially convex in the Jensen sense. Also, using basic convexity theory, it follows that a positive function is log-convex if and only if it is 2-exponentially convex.

The following lemma is equivalent to the definition of convex function (see [6, page 2]).

**Lemma 38 .**

If *x*_1_, *x*_2_, *x*_3_ ∈ *I* are such that *x*_1_ < *x*_2_ < *x*_3_, then the function *f* : *I* → *ℝ* is convex if and only if the following inequality holds:

(59) $$\begin{array}{c} \left(x_{3}-x_{2}\right)f\left(x_{1}\right)+\left(x_{1}-x_{3}\right)f\left(x_{2}\right)+\left(x_{2}-x_{1}\right)f\left(x_{3}\right)\geq 0. \end{array}$$

We will also need the following result (see, e.g., [6]).

**Proposition 39 .**

If *f* : *I* → *ℝ* is a convex function and *x*_1_, *x*_2_, *y*_1_, *y*_2_ ∈ *I* are such that *x*_1_ ≤ *y*_1_, *x*_2_ ≤ *y*_2_, *x*_1_ ≠ *x*_2_, and *y*_1_ ≠ *y*_2_, then the following inequality is valid:

(60) $$\begin{array}{c} \frac{f\left(x_{2}\right)-f\left(x_{1}\right)}{x_{2}-x_{1}}\leq \frac{f\left(y_{2}\right)-f\left(y_{1}\right)}{y_{2}-y_{1}}. \end{array}$$

When dealing with functions with different degree of smoothness, divided differences are found to be very useful.

**Definition 40 .**

The second order divided difference of a function *f* : *I* → *ℝ* at mutually different points *x*_0_,   *x*_1_,   *x*_2_ ∈ *I* is defined recursively by

(61) $$\begin{array}{c} \left[x_{i};f\right]=f\left(x_{i}\right),\;i=0,1,2,\\ \left[x_{i},x_{i+1};f\right]=\frac{f\left(x_{i+1}\right)-f\left(x_{i}\right)}{x_{i+1}-x_{i}},\;i=0,1,\\ \left[x_{0},x_{1},x_{2};f\right]=\frac{\left[x_{1},x_{2};f\right]-\left[x_{0},x_{1};f\right]}{x_{2}-x_{0}}. \end{array}$$

**Remark 41 .**

The value [*x*_0_, *x*_1_, *x*_2_; *f*] is independent of the order of the points *x*_0_, *x*_1_, and *x*_2_. This definition may be extended to include the case in which some or all the points coincide (see [6, page 16]). Namely, taking the limit *x*_1_ → *x*_0_ in (61), we obtain

(62) lim⁡x1→x0[x0,x1,x2;f]=[x0,x0,x2;f]=f(x2)−f(x0)−f′(x0)(x2−x0)(x2−x0)2,aaaaaaaaaaaaaaaaaaaaaaaaax2≠x0

provided that *f*′ exists, and furthermore, taking the limits *x*_*i*_ → *x*_0_, *i* = 1,2, in (61), we obtain

(63) $$\begin{array}{c} \underset{x_{2}\to x_{0}}{\lim}\;\underset{x_{1}\to x_{0}}{\lim}\left[x_{0},x_{1},x_{2};f\right]=\left[x_{0},x_{0},x_{0};f\right]=\frac{f^{\prime \prime }\left(x_{0}\right)}{2} \end{array}$$

provided that *f*′′ exists.

A function *f* : *I* → *ℝ* is convex if and only if for every choice of three mutually different points *x*_0_, *x*_1_, *x*_2_ ∈ *I*[*x*_0_, *x*_1_, *x*_2_; *f*] ≥ 0 holds.

Now, we use an idea from [15] to give an elegant method of producing an *n*-exponentially convex and exponentially convex functions applying the functionals *𝒥*_1_ and *𝒥*_2_ on a given family of functions with the same property.

**Theorem 42 .**

Let *𝒥*_*i*_, *i* = 1,2, be linear functionals defined in (26) and (50), respectively. Let *Ω* = {Φ_*ρ*_ : *ρ* ∈ *J*}, where *J* is an interval in *ℝ*, be a family of functions Φ_*ρ*_ ∈ *C*([*m*, *M*], *ℝ*) such that the function *ρ* ↦ [*x*_0_, *x*_1_, *x*_2_; Φ_*ρ*_] is *n*-exponentially convex in the Jensen sense on *J* for every choice of three mutually different points *x*_0_, *x*_1_, *x*_2_ ∈ [*m*, *M*]. Then *ρ* ↦ *𝒥*_*i*_(*g*, Φ_*ρ*_) is an *n*-exponentially convex function in the Jensen sense on *J*. If the function *ρ* ↦ *𝒥*_*i*_(*g*, Φ_*ρ*_) is also continuous on *J*, then it is *n*-exponentially convex on *J*.

**Proof**

Define the function *ν* : *I* → *ℝ* by

(64) $$\begin{array}{c} \nu \left(x\right)=\overset{n}{\underset{j,k=1}{\sum }}\xi_{j}\xi_{k}\Phi_{r_{jk}}\left(x\right), \end{array}$$

where *ξ*_*j*_, *ξ*_*k*_ ∈ *ℝ*, *r*_*j*_, *r*_*k*_ ∈ *J*, 1 ≤ *j*, *k* ≤ *n*, *r*_*jk*_ = (*r*_*j*_ + *r*_*k*_)/2, and Φ_*r*_*jk*__ ∈ *Ω*. Using the assumption that for every choice of three mutually different points *x*_0_, *x*_1_, *x*_2_ ∈ [*m*, *M*]*ρ* ↦ [*x*_0_, *x*_1_, *x*_2_; Φ_*ρ*_] is *n*-exponentially convex in the Jensen sense on *J*, we obtain

(65) $$\begin{array}{c} \left[x_{0},x_{1},x_{2};\nu \right]=\overset{n}{\underset{j,k=1}{\sum }}\xi_{j}\xi_{k}\left[x_{0},x_{1},x_{2};\Phi_{r_{jk}}\right]\geq 0. \end{array}$$

Therefore, *ν* is convex (and continuous) function on *I*. Hence *𝒥*_*i*_(*g*, *ν*) ≥ 0, *i* = 1,2, which implies that

(66) $$\begin{array}{c} \overset{n}{\underset{j,k=1}{\sum }}\xi_{j}\xi_{k}J_{i}\left(g,\Phi_{r_{jk}}\right)\geq 0. \end{array}$$

We conclude that the function *ρ* ↦ *𝒥*_*i*_(*g*, Φ_*ρ*_) is *n*-exponentially convex on *J* in the Jensen sense.

If *ρ* ↦ *𝒥*_*i*_(*g*, Φ_*ρ*_) is continuous on *J*, then *ρ* ↦ *𝒥*_*i*_(*g*, Φ_*ρ*_) is *n*-exponentially convex by definition.

The following corollary is an immediate consequence of Theorem 42.

**Corollary 43 .**

Let *𝒥*_*i*_, *i* = 1,2, be linear functionals defined in (26) and (50), respectively. Let *Ω* = {Φ_*ρ*_ : *ρ* ∈ *J*}, where *J* is an interval in *ℝ*, be a family of functions Φ_*ρ*_ ∈ *C*([*m*, *M*], *ℝ*) such that the function *ρ* ↦ [*x*_0_, *x*_1_, *x*_2_; Φ_*ρ*_] is exponentially convex in the Jensen sense on *J* for every choice of three mutually different points *x*_0_, *x*_1_, *x*_2_ ∈ [*m*, *M*]. Then *ρ* ↦ *𝒥*_*i*_(*g*, Φ_*ρ*_) is an exponentially convex function in the Jensen sense on *J*. If the function *ρ* ↦ *𝒥*_*i*_(*g*, Φ_*ρ*_) is also continuous on *J*, then it is exponentially convex on *J*.

**Corollary 44 .**

Let *𝒥*_*i*_, *i* = 1,2, be linear functionals defined in (26) and (50), respectively. Let *Ω* = {Φ_*ρ*_ : *ρ* ∈ *J*}, where *J* is an interval in *ℝ*, be a family of functions Φ_*ρ*_ ∈ *C*([*m*, *M*], *ℝ*) such that the function *ρ* ↦ [*x*_0_, *x*_1_, *x*_2_; Φ_*ρ*_] is 2-exponentially convex in the Jensen sense on *J* for every choice of three mutually different points *x*_0_, *x*_1_, *x*_2_ ∈ [*m*, *M*]. Then, the following statements hold.

(i) *ρ* ↦ *𝒥*_*i*_(*g*, Φ_*ρ*_) is a 2-exponentially convex function in the Jensen sense on *J*.
(ii) If *ρ* ↦ *𝒥*_*i*_(*g*, Φ_*ρ*_) is continuous on *J*, then it is also 2-exponentially convex on *J*. If *ρ* ↦ *𝒥*_*i*_(*g*, Φ_*ρ*_) is additionally strictly positive, then it is also log-convex on *J*, and for *r*, *s*, *t* ∈ *J* such that *r* < *s* < *t*, we have
  (67) $$\begin{array}{c} {\left(J_{i}(g,\Phi_{s})\right)}^{t-r}\leq {\left(J_{i}(g,\Phi_{r})\right)}^{t-s}{\left(J_{i}(g,\Phi_{t})\right)}^{s-r}. \end{array}$$
(iii) If *ρ* ↦ *𝒥*_*i*_(*g*, Φ_*ρ*_) is strictly positive and differentiable function on *J*, then for every *p*, *q*, *u*, *v* ∈ *J* such that *p* ≤ *u*, *q* ≤ *v*, we have
  (68) $$\begin{array}{c} M_{p,q}\left(g,J_{i},\Omega \right)\leq M_{u,v}\left(g,J_{i},\Omega \right),\;i=1,2, \end{array}$$
  where
  (69) $$\begin{array}{c} M_{p,q}\left(g,J_{i},\Omega \right)=\{\begin{array}{cc} {\left(\frac{J_{i}(g,\Phi_{p})}{J_{i}(g,\Phi_{q})}\right)}^{1/\left(p-q\right)}, & p\neq q;\\ \exp\left(\frac{\left(d/dp\right)J_{i}\left(g,\Phi_{p}\right)}{J_{i}\left(g,\Phi_{p}\right)}\right), & p=q, \end{array} \end{array}$$
  for Φ_*p*_, Φ_*q*_ ∈ *Ω*.

**Proof**

(i) and the first part of (ii) are immediate consequences of Theorem 42. If *ρ* ↦ *𝒥*_*i*_(*g*, Φ_*ρ*_) is continuous and strictly positive, its log-convexity is an immediate consequence of Remark 37. Now applying Lemma 38 on the function *f*(*x*) = log⁡*𝒥*_*i*_(*g*, Φ_*x*_) and *r*, *s*, *t* ∈ *J* (*r* < *s* < *t*), we get

(70) $$\begin{array}{c} \left(t-s\right)J_{i}\left(g,\Phi_{r}\right)+\left(r-t\right)J_{i}\left(g,\Phi_{s}\right)+\left(s-r\right)J_{i}\left(g,\Phi_{t}\right)\geq 0, \end{array}$$

which is equivalent to inequality (67).

To prove (iii), let *ρ* ↦ *𝒥*_*i*_(*g*, Φ_*ρ*_) be strictly positive and differentiable and therefore continuous too. By (ii), the function *ρ* ↦ *𝒥*_*i*_(*g*, Φ_*ρ*_) is log-convex on *J*; that is, the function *ρ* ↦ log⁡*𝒥*_*i*_(*g*, Φ_*ρ*_) is convex on *J*, and by Proposition 39 we obtain

(71) log⁡Ji(g,Φp)−log⁡Ji(g,Φq)p−q ≤log⁡Ji(g,Φu)−log⁡Ji(g,Φv)u−v

for *p* ≤ *u*, *q* ≤ *v*, *p* ≠ *q*, and *u* ≠ *v*, concluding that

(72) $$\begin{array}{c} M_{p,q}\left(g,J_{i},\Omega \right)\leq M_{u,v}\left(g,J_{i},\Omega \right). \end{array}$$

The cases *p* = *q* and *u* = *v* follow from (71) as limit cases.

**Remark 45 .**

Note that the results from Theorem 42, Corollary 43, and Corollary 44 still hold when two of the points *x*_0_, *x*_1_, *x*_2_ ∈ [*m*, *M*] coincide, for a family of differentiable functions Φ_*ρ*_ such that the function *ρ* ↦ [*x*_0_, *x*_1_, *x*_2_; Φ_*ρ*_] is *n*-exponentially convex in the Jensen sense (exponentially convex in the Jensen sense and log-convex in the Jensen sense), and furthermore, they still hold when all three points coincide for a family of twice differentiable functions with the same property. The proofs are obtained by recalling Remark 41 and suitable characterization of convexity.

## 5. Examples

In this section we will vary on choice of a family *Ω* = {Φ_*ρ*_ : *ρ* ∈ *J*} in order to construct different examples of exponentially convex functions and construct some means.

**Example 46 .**

Consider a family of functions

(73) $$\begin{array}{c} \Omega_{1}=\left\{\kappa_{\rho }:R⟶\left[0,\infty \right);\rho \in R\right\} \end{array}$$

defined by

(74) $$\begin{array}{c} \kappa_{\rho }\left(x\right)=\{\begin{array}{cc} \frac{1}{\rho^{2}}e^{\rho x}, & \rho \neq 0;\\ \frac{1}{2}x^{2}, & \rho =0. \end{array} \end{array}$$

It is (*d* ^2^/*dx* ^2^)*κ*_*ρ*_(*x*) = *e* ^*ρx*^ > 0 which shows that *κ*_*ρ*_ is convex on *ℝ* for every *ρ* ∈ *ℝ*. From Remark 36, it follows that *ρ* ↦ (*d* ^2^/*dx* ^2^)*κ*_*ρ*_(*x*) is exponentially convex. Therefore, *ρ* ↦ [*x*_0_, *x*_1_, *x*_2_; *κ*_*ρ*_] is exponentially convex (see [15]) (and so exponentially convex in the Jensen sense). Now using Corollary 43 we conclude that *ρ* ↦ *𝒥*_*i*_(*g*, *κ*_*ρ*_), (*i* = 1,2) are exponentially convex in the Jensen sense. It is easy to verify that these mappings are continuous, so they are exponentially convex.

For this family of functions, *ℳ*_*p*,*q*_(*g*, *𝒥*_*i*_, *Ω*) from (69) becomes

(75) Mp,q(g,Ji,Ω1) ={(Ji(g,κp)Ji(g,κq))1/(p−q),p≠q;exp⁡(Ji(g,id·κp)Ji(g,κp)−2p),p=q≠0;exp⁡(Ji(g,id·κ0)3Ji(g,κ0)),p=q=0,

and using (68) we have that it is monotonous in parameters *p* and *q*.

If *𝒥*_*i*_  (*i* = 1,2) are positive, using Theorems 18 and 31 applied for Φ = *κ*_*p*_ ∈ *Ω*_1_ and Ψ = *κ*_*q*_ ∈ *Ω*_1_, it follows that

(76) $$\begin{array}{c} {ℵ}_{p,q}\left(g,J_{i},\Omega_{1}\right)=\log M_{p,q}\left(g,J_{i},\Omega_{1}\right)\;\left(i=1,2\right) \end{array}$$

satisfy *ℵ*_*p*,*q*_(*g*, *𝒥*_*i*_, *Ω*_1_)∈[*m*, *M*]. If we set *g*([*a*, *b*]_*𝕋*_) = [*m*, *M*] then we have that *ℵ*_*p*,*q*_(*g*, *𝒥*_*i*_, *Ω*_1_) are means (of the function *g*). Note that by (68) they are monotonous means.

**Example 47 .**

Consider a family of functions

(77) $$\begin{array}{c} \Omega_{2}=\left\{\beta_{\rho }:\left(0,\infty \right)⟶R;\rho \in R\right\} \end{array}$$

defined by

(78) $$\begin{array}{c} \beta_{\rho }\left(x\right)=\{\begin{array}{cc} \frac{x^{\rho }}{\rho \left(\rho -1\right)}, & \rho \neq 0,1;\\ -\log x, & \rho =0;\\ x\log x, & \rho =1. \end{array} \end{array}$$

It is (*d* ^2^/*dx* ^2^)*β*_*ρ*_(*x*) = *x* ^*ρ*−2^ = *e* ^(*ρ*−2)log⁡*x*^ > 0, which shows that *β*_*ρ*_ is convex function for *x* > 0. Also, from Remark 36 it follows that *ρ* ↦ (*d* ^2^/*dx* ^2^)*β*_*ρ*_(*x*) is exponentially convex. Therefore *ρ* ↦ [*x*_0_, *x*_1_, *x*_2_; *β*_*ρ*_] is exponentially convex (and so exponentially convex in the Jensen sense). Here we assume that [*m*, *M*]⊂(0, *∞*), so our family *Ω*_2_ of *β*_*ρ*_ fulfills the conditions of Corollary 43. In this case *ℳ*_*p*,*q*_(*g*, *𝒥*_*i*_, *Ω*) from (69) becomes

(79) Mp,q(g,Ji,Ω2) ={(Ji(g,βp)Ji(g,βq))1/(p−q),p≠q;exp⁡(1−2pp(p−1)−Ji(g,β0βp)Ji(g,βp)),p=q≠0,1;exp⁡(1−Ji(g,β02)2Ji(g,β0)),p=q=0;exp⁡(−1−Ji(g,β0β1)2Ji(g,β1)),p=q=1.

If *𝒥*_*i*_  (*i* = 1,2) are positive, by applying Theorems 18 and 31 for Φ = *β*_*p*_ ∈ *Ω*_2_ and Ψ = *β*_*q*_ ∈ *Ω*_2_, it follows that for *i* = 1,2 there exist *ξ*_*i*_ ∈ [*m*, *M*] such that

(80) $$\begin{array}{c} \xi_{\text{i}}^{p-q}=\frac{J_{i}\left(g,\beta_{p}\right)}{J_{i}\left(g,\beta_{q}\right)}. \end{array}$$

Since the function *ξ*_*i*_ ↦ *ξ*_*i*_^*p*−*q*^ is invertible for *p* ≠ *q*, we have

(81) $$\begin{array}{c} m\leq {\left(\frac{J_{i}(g,\beta_{p})}{J_{i}(g,\beta_{q})}\right)}^{1/\left(p-q\right)}\leq M. \end{array}$$

Also, *ℳ*_*p*,*q*_(*g*, *𝒥*_*i*_, *Ω*_2_) is continuous, symmetric, and monotonous (by (68)). If we set *g*([*a*, *b*]_*𝕋*_) = [*m*, *M*], then we have that

(82) m=min⁡t∈[a,b]T{g(t)}≤(Ji(g,βp)Ji(g,βq))1/(p−q)≤max⁡t∈[a,b]T{g(t)}=M,aaaaaaaaifor  i=1,2,

which shows that *ℳ*_*p*,*q*_(*g*, *𝒥*_*i*_, *Ω*_2_) are means (of the function *g*).

Now we impose one additional parameter *r* in case *g*([*a*, *b*]_*𝕋*_) = [*m*, *M*]. For *r* ≠ 0 by substituting *g* ↦ *g* ^*r*^, *p* ↦ *p*/*r*, and *q* ↦ *q*/*r* in (82), we get

(83) m=min⁡t∈[a,b]T{gr(t)}≤(Ji(gr,βp)Ji(gr,βq))r/(p−q)≤max⁡t∈[a,b]T{gr(t)}=M,aaaaaaaaaifor  i=1,2.

We define new generalized means as follows:

(84) $$\begin{array}{c} M_{p,q;r}\left(g,J_{i},\Omega_{2}\right)=\{\begin{array}{cc} {\left(M_{p/r,q/r}(g^{r},J_{i},\Omega_{2})\right)}^{1/r}, & r\neq 0;\\ \left(M_{p/r,q/r}\left(\log g,J_{i},\Omega_{1}\right)\right), & r=0. \end{array} \end{array}$$

These new generalized means are also monotonic.

**Example 48 .**

Consider a family of functions

(85) $$\begin{array}{c} \Omega_{3}=\left\{\gamma_{\rho }:\left(0,\infty \right)⟶\left(0,\infty \right):\rho \in \left(0,\infty \right)\right\} \end{array}$$

defined by

(86) $$\begin{array}{c} \gamma_{\rho }\left(x\right)=\{\begin{array}{cc} \frac{\rho^{-x}}{{\left(\log\rho \right)}^{2}}, & \rho \neq 1;\\ \frac{x^{2}}{2}, & \rho =1. \end{array} \end{array}$$

It is (*d* ^2^/*dx* ^2^)*γ*_*ρ*_(*x*) = *ρ* ^−*x*^ > 0, which shows that *γ*_*ρ*_ is convex function for *ρ* > 0. Also, from Remark 36 it follows that *ρ* ↦ (*d* ^2^/*dx* ^2^)*γ*_*ρ*_(*x*) is exponentially convex. Therefore *ρ* ↦ [*x*_0_, *x*_1_, *x*_2_; *γ*_*ρ*_] is exponentially convex (and so exponentially convex in the Jensen sense). Here we assume that [*m*, *M*]⊂(0, *∞*), so our family *Ω*_3_ of *γ*_*ρ*_ fulfills the conditions of Corollary 43. In this case *ℳ*_*p*,*q*_(*g*, *𝒥*_*i*_, *Ω*) from (69) becomes

(87) Mp,q(g,Ji,Ω3) ={(Ji(g,γp)Ji(g,γq))1/(p−q),p≠q;exp⁡(−Ji(g,id·γp)pJi(g,γp)−2plog⁡p),p=q≠1;exp⁡(−2Ji(g,id·γ1)3Ji(g,γ1)),p=q=1,

and by (68) it is monotonous function in parameters *p* and *q*.

If *𝒥*_*i*_  (*i* = 1,2) are positive, using Theorems 18 and 31 applied for Φ = *γ*_*p*_ ∈ *Ω*_3_ and Ψ = *γ*_*q*_ ∈ *Ω*_3_, it follows that

(88) $$\begin{array}{c} {ℵ}_{p,q}\left(g,J_{i},\Omega_{3}\right)=-L\left(p,q\right)\log M_{p,q}\left(g,J_{i},\Omega_{3}\right) \end{array}$$

satisfies *ℵ*_*p*,*q*_(*g*, *𝒥*_*i*_, *Ω*_3_)∈[*m*, *M*]. Here *L*(*p*, *q*) is the logarithmic mean defined by *L*(*p*, *q*) = (*p* − *q*)/(log⁡*p* − log⁡*q*) for *p* ≠ *q*, *L*(*p*, *p*) = *p*.

**Example 49 .**

Consider a family of functions

(89) $$\begin{array}{c} \Omega_{4}=\left\{\delta_{\rho }:\left(0,\infty \right)⟶\left(0,\infty \right):\rho \in \left(0,\infty \right)\right\} \end{array}$$

defined by

(90) $$\begin{array}{c} \delta_{\rho }\left(x\right)=\frac{e^{-x\sqrt{\rho }}}{\rho }. \end{array}$$

It is $(d^{2}/dx^{2})\delta_{\rho }(x)=e^{-x\sqrt{\rho }}>0$, which shows that *δ*_*ρ*_ is convex function for *ρ* > 0. Also, from Remark 36 it follows that *ρ* ↦ (*d* ^2^/*dx* ^2^)*δ*_*ρ*_(*x*) is exponentially convex. Therefore *ρ* ↦ [*x*_0_, *x*_1_, *x*_2_; *δ*_*ρ*_] is exponentially convex (and so exponentially convex in the Jensen sense). Here we assume that [*m*, *M*]⊂(0, *∞*), so our family *Ω*_4_ of *δ*_*ρ*_ fulfills the conditions of Corollary 43. In this case *ℳ*_*p*,*q*_(*g*, *𝒥*_*i*_, *Ω*) from (69) becomes

(91) Mp,q(g,Ji,Ω4) ={(Ji(g,δp)Ji(g,δq))1/(p−q),p≠q;exp⁡(−Ji(g,id·δp)2pJi(g,δp)−1p),p=q,

and it is monotonous function in parameters *p* and *q* by (68).

If *𝒥*_*i*_  (*i* = 1,2) are positive, using Theorems 18 and 31 applied for Φ = *δ*_*p*_ ∈ *Ω*_4_ and Ψ = *δ*_*q*_ ∈ *Ω*_4_, it follows that

(92) $$\begin{array}{c} {ℵ}_{p,q}\left(g,J_{i},\Omega_{4}\right)=-\left(\sqrt{p}+\sqrt{q}\right)\log M_{p,q}\left(g,J_{i},\Omega_{4}\right) \end{array}$$

satisfies *ℵ*_*p*,*q*_(*g*, *𝒥*_*i*_, *Ω*_4_)∈[*m*, *M*].

## References

[1] Sheng Q., Fadag M., Henderson J., Davis J. M. (2006). An exploration of combined dynamic derivatives on time scales and their applications. *Nonlinear Analysis: Real World Applications*.

[2] Ammi M. R. S., Ferreira R. A. C., Torres D. F. M. (2008). Diamond-*α* Jensen's inequality on time scales. *Journal of Inequalities and Applications*.

[3] Anwar M., Bibi R., Bohner M., Pečarić J. (2011). Integral inequalities on time scales via the theory of isotonic linear functionals. *Abstract and Applied Analysis*.

[4] Dinu C. (2009). A weighted Hermite Hadamard inequality for Steffensen-Popoviciu and Hermite-Hadamard weights on time scales. *Analele Ştiinţifice ale Universităţii “Ovidius” Constanţa. Seria: Matematică*.

[5] Steffensen J. F. (1919). On certain inequalities and methods of approximation. *Journal of the Institute of Actuaries*.

[6] Pečarić J. E., Proschan F., Tong Y. L. (1992). *Convex Functions, Partial Orderings, and Statistical Applications*.

[7] Boas, R. P. (1970). The Jensen-Steffensen inequality. *Univerzitet u Beogradu. Publikacije Elektrotehničkog Fakulteta. Serija Matematika i Fizika*.

[8] Pečarić J., Perić I., Rodić Lipanović M. (2014). Uniform treatment of Jensen type inequalities. *Mathematical Reports*.

[9] Khan A. R., Pečarić J., Rodić Lipanović M. (2013). n-exponential convexity for Jensen-type inequalities. *Journal of Mathematical Inequalities*.

[10] Niculescu C. P., Persson L.-E. (2006). *Convex Functions and Their Applications: A Contemporary Aproach*.

[11] Widder D. V. (1942). Completely convex functions and Lidstone series. *Transactions of the American Mathematical Society*.

[12] Franjić I., Khalid S., Pečarić J. (2011). On the refinements of Jensen-Steffensen inequalities. *Journal of Inequalities and Applications*.

[13] Klaričić Bakula M., Pečarić J., Perić J. (2012). Extensions of the Hermite-Hadamard inequality with applications. *Mathematical Inequalities & Applications*.

[14] Pečarić J., Perić J. (2012). Improvements of the Giaccardi and the Petrović inequality and related Stolarsky type means. *Analele Universităţii din Craiova. Seria Matematică Informatică*.

[15] Jakšetić J., Pečarić J. (2013). Exponential Convexity Method. *Journal of Convex Analysis*.

